# Supplementary material for: Genome-Wide Identification, Characterization, and Expression Analysis of Four Subgroup Members of the GH13 Family in Wheat (Triticum aestivum L.)
Source: Int J Mol Sci. 2024 Mar 17;25(6):3399. doi: 10.3390/ijms25063399 (PMC10970197; doi:10.3390/ijms25063399)
Supplement: Supplementary file 1 [file ijms-25-03399-s001.zip › SUPPLEMENTARY MATERIAL Figures.pdf]

SUPPLEMENTARY MATERIAL Figures:

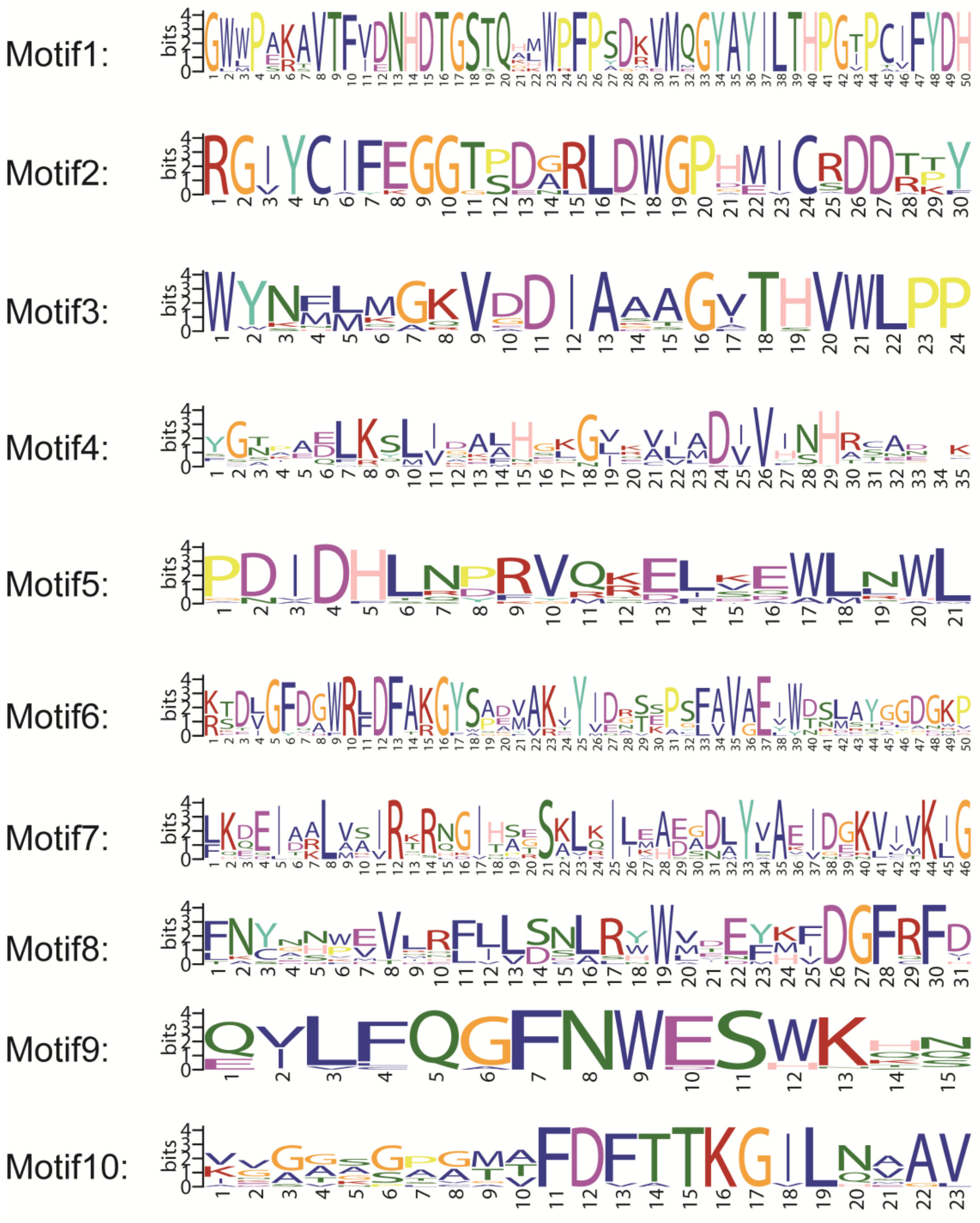

Figure S1. The 10 motif logos detected by MEME database demonstrate the possible amino acid sequences that make up each motif.

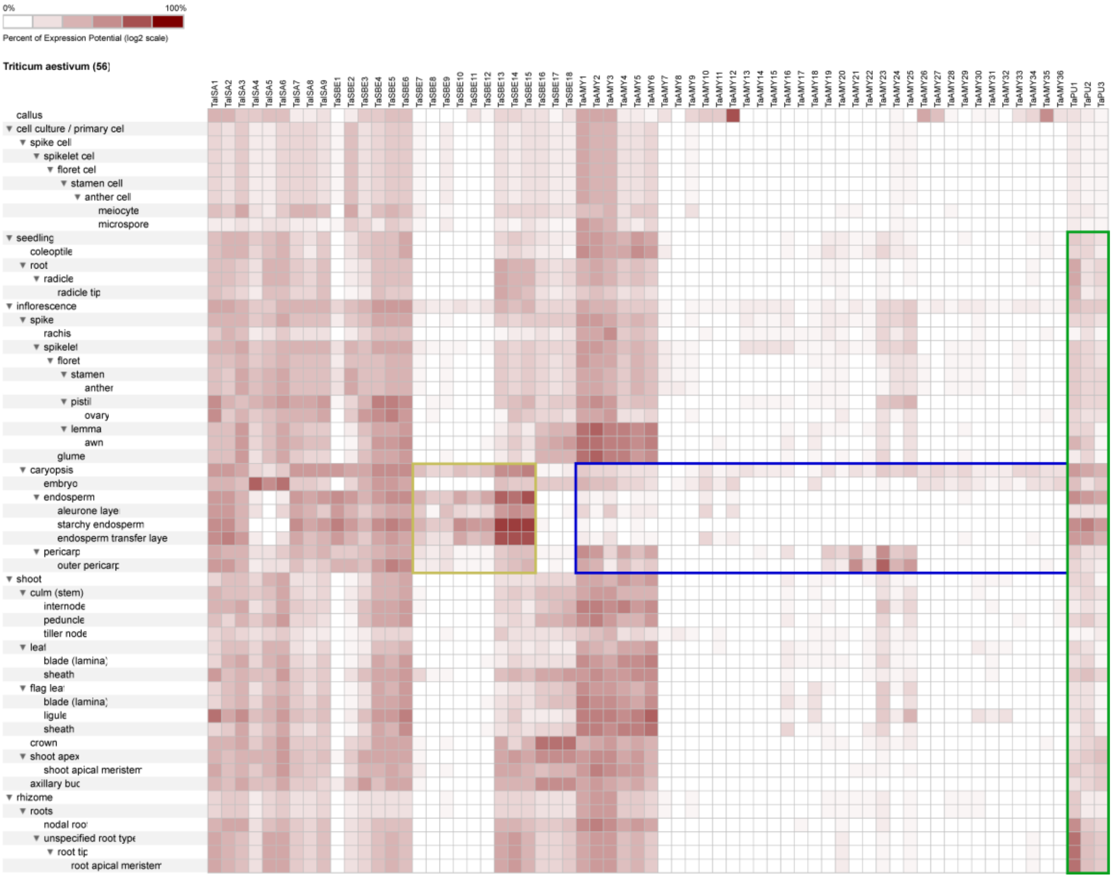

Figure S2. Differences in TaGH13 expression levels in different tissues. The expression of all 66 genes in different tissues is shown in the graphs and filled with light to dark red according to the expression from low to high. The selected area of yellow squares indicates the expression of *TaSBE7-15* in seeds. The selected area of blue squares indicates the expression of *TaAMY* in seeds. The selected area of green squares indicates the expression of *TaPU* in different tissues of the whole plant.
